# Supplementary material for: A Critical View of the Application of the APEX Software (Aqueous Photochemistry of Environmentally-Occurring Xenobiotics) to Predict Photoreaction Kinetics in Surface Freshwaters
Source: Molecules. 2019 Dec 18;25(1):9. doi: 10.3390/molecules25010009 (PMC7017383; doi:10.3390/molecules25010009)
Supplement: Supplementary file 1 [file molecules-25-00009-s001.zip › 3.2.4_gcc-4.4.0.zip!/3.2.4_gcc-4.4.0/3.2.4_gcc-4.4.0/doc/octave/html/interpreter/Promotion-and-Demotion-of-Data-Types.html]

Promotion and Demotion of Data Types - Untitled


Next: Predicates for Numeric Objects,
Previous: Logical Values,
Up: Numeric Data Types


---

### 4.7 Promotion and Demotion of Data Types

Many operators and functions can work with mixed data types. For example

```
     uint8 (1) + 1
         => 2
```

where the above operator works with an 8-bit integer and a double precision
value and returns an 8-bit integer value. Note that the type is demoted
to an 8-bit integer, rather than promoted to a double precision value as
might be expected. The reason is that if Octave promoted values in
expressions like the above with all numerical constants would need to be
explicitly cast to the appropriate data type like

```
     uint8 (1) + uint8 (1)
         => 2
```

which becomes difficult for the user to apply uniformly and might allow
hard to find bugs to be introduced. The same applies to single precision
values where a mixed operation such as

```
     single (1) + 1
         => 2
```

returns a single precision value. The mixed operations that are valid
and their returned data types are

|  |  |  |  |
| --- | --- | --- | --- |
|  | Mixed Operation | Result |  |
|  | double OP single | single |  |
|  | double OP integer | integer |  |
|  | double OP char | double |  |
|  | double OP logical | double |  |
|  | single OP integer | integer |  |
|  | single OP char | single |  |
|  | single OP logical | single |  |

The same logic applies to functions with mixed arguments such as

```
     min (single (1), 0)
        => 0
```

where the returned value is single precision.

In the case of mixed type indexed assignments, the type is not
changed. For example

```
     x = ones (2, 2);
     x (1, 1) = single (2)
         => x = 2   1
                1   1
```

where `x` remains of the double precision type.
